# The Development and Validation of a Nomogram Incorporating Clinical, Pathological, and Therapeutic Features to Predict Overall Survival in Patients With Penile Cancer: A SEER-Based Study

**DOI:** 10.3389/fonc.2022.840367

**Published:** 2022-04-05

**Authors:** Ruidan Li, Ke Cheng, Zhigong Wei, Zheran Liu, Xingchen Peng

**Affiliations:** ^1^Department of Biotherapy, Cancer Center, the State Key Laboratory of Biotherapy, West China Hospital, West China Medical School, Sichuan University, Chengdu, China; ^2^Department of Abdominal Oncology, Cancer Center, West China Hospital, Sichuan University, Chengdu, China

**Keywords:** penile cancer, prognostic factors, overall survival, nomogram, overall survival

## Abstract

**Objective:**

This study aimed to investigate the prognostic factors of penile cancer and establish a comprehensive predictive model for clinical application.

**Methods:**

A total of 581 patients from the Surveillance, Epidemiology, and End Results (SEER) program (2000–2018) were used to develop the prognostic model. The multivariate Cox proportional hazards regression was performed to identify independent prognostic factors to develop the nomogram. The performance of this model was validated internally by a cohort with 143 patients from the SEER database and validated externally by a cohort with 70 patients from the West China Hospital, Sichuan University (2010–2020).

**Results:**

Age, marital status, size of the primary lesion, primary tumor (T), regional lymph nodes status, distant metastasis (M), and the surgery of regional lymph node (LND) were the independent prognostic factors for overall survival (OS) and were incorporated in the prognostic model. The prognostic nomogram showed a good risk stratification ability for OS in the development cohort, internal validation cohort, and external validation cohort.

**Conclusion:**

This study incorporates the clinical, pathological, and therapeutic features comprehensively to develop a novel and clinically effective prognostic model for patients with penile cancer.

## Introduction

Penile cancer is a rare disease (1); the incidence of penile cancer in 2020 was approximately 2,200 cases in the United States (2). However, advanced penile cancer is associated with considerable morbidity and mortality (3). Many patients cannot preserve organ function by conservative therapy due to the advanced stages of the disease (4).

Risk factors for penile cancer include the absence of childhood circumcision, phimosis, chronic inflammation, poor penile hygiene, smoking, immunosuppression, and infection with human papillomavirus (HPV) (5). Additionally, the HPV infection has been proven to be not only associated with the incidence of penile cancer but also related to the prognosis of patients with penile cancer (6, 7). In Lont’s study, disease-specific 5-year survival in the high-risk HPV-negative group and high-risk HPV-positive group was 78% and 93%, respectively (7). Additionally, in many studies, the lymph node status was considered to be the strongest prognostic factor for penile cancer (5, 8–10). Moreover, with the widespread use of immunotherapy, the prognostic role of PD-L1 in penile cancer has also attracted attention. De Bacco et al. analyzed retrospectively 40 patients with penile squamous cell carcinoma; 18 (51.4%) patients were PD-L1-positive, and PD-L1 expression appears to be associated with larger tumors and worse clinical outcomes (11). Findings in other studies also confirmed the prognostic role of PD-L1 (12, 13).

Organ-preservation strategies were justified for patients with early primary disease, and partial or total penectomy was considered as the gold standard treatment for the advanced primary disease with a low local recurrence rate (14). According to the limited studies, prophylactic inguinal lymphadenectomy (ILND) showed superior survival in clinically node-negative patients compared with radiotherapy or surveillance (15). Radical ILND was preferred when positive nodes were clinically detected (16). In addition, previous evidence suggested that adjuvant chemotherapy might bring survival benefits to patients with positive lymph nodes (17). However, adjuvant radiotherapy has not been proven useful after surgery with poor evidence (16, 18). Until now, optimal treatment mode is unclear, because of the lack of large series and controlled trials.

Some prognostic models have been developed to predict survival in patients with penile cancer. For instance, Kattan MW et al. built two nomograms based on the clinical and pathological data for predicting survival in patients undergoing partial or total amputation (19). However, the sample size used to develop models in Kattan’s study was relatively small (175 patients). Although there were other prognostic models based on the relatively larger samples for penile cancer, the vital step of model construction with external validation of these models was lacking. In the present study, we established and validated a prognostic model for patients with penile cancer.

## Materials and Methods

### Patients’ Inclusion

**Figure 1:**
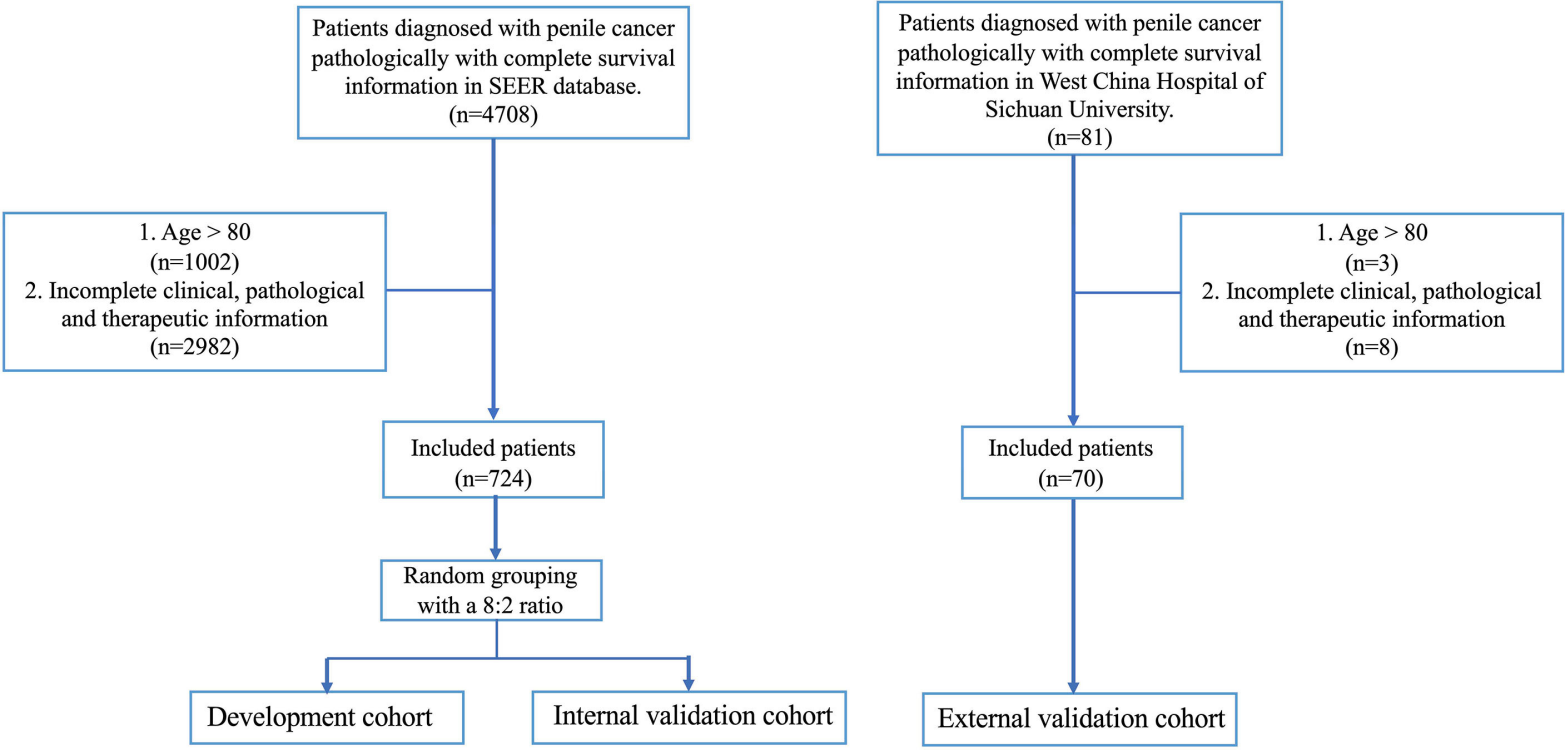
Flow chart of study design.

The patients of development and internal validation cohort came from the SEER program of the National Cancer Institute (http://seer.cancer.gov/) and the external validation cohort came from West China Hospital of Sichuan University (from 2010 to 2020). The inclusion criteria were as follows (a) Penile cancer was the first and only primary diagnosis, (b) The diagnosis of penile cancer was identified by pathological examination, (c) Follow-up data of patients could be attained completely. The exclusion criteria were as follows (a) Age more than 80 years old, (b) The required clinical, pathological, and therapeutic information was incomplete (**Figure 1**).

### Data Collection and Endpoint Definition

The relevant data collection of all eligible cases included age at diagnosis, marital status, race, the site of the primary lesion, histologic type, differentiation grade, size of the primary lesion, the information of stage (T, N, M and clinical stage), the information of the treatment, and survival outcomes. The marital status of patients was classified into “married” and “other” groups. Patients who were “separated”, “single (never married)”, “widowed”, “divorced”, and “unmarried or domestic partners” were in the group of “other”. The primary site included C60.0 (prepuce), C60.1 (glans penis), C60.2 (body of penis), C60.8 (overlapping lesion of penis), and C60.9 (penis NOS) according to the International Classification of Diseases for Oncology, Third Edition (ICD-O-3). The information of stage was determined by the American Joint Committee on Cancer (AJCC) TNM Staging System for Penile Cancer (7th ed., 2010). The treatment-related data included the primary surgery, the surgery of regional lymph node (LND), radiotherapy, and chemotherapy. The LND was determined by the variables “RX Summ-Scope Reg LN Sur (2003+)” in the SEER database.

The primary endpoint was OS. It was measured from the date of initial diagnosis of penile cancer to the date of death from any cause.

### Statistical Analysis

The categorical variables were presented by frequencies and proportions and tested by the Chi-square test. Multivariate Cox proportional hazards model was performed to select the independent prognostic factors of OS. Finally, these independent prognostic factors were included to develop the model for predicting the 3- and 5-year OS rates in patients with penile cancer.

The prognostic model was validated internally by a cohort with 143 patients from SEER database and validated externally by using a cohort from West China Hospital. External validation was a crucial step to determine the reproducibility of a predictive model and its generalizability to new and different patients (20). Discrimination of one model was characterized by the ability it differentiates those at higher risk of having an event from those at lower risk (21). The receiver operating characteristic (ROC) curve was used to evaluate the discrimination of model in the current study, and the higher area under the ROC curve (AUC) means the better model (22). The calibration plots in this study were used to exhibit the accordance between predicted survival and actual survival (21). Furthermore, the decision curve analysis (DCA) was conducted to compare the net benefits and clinical effectiveness of this model with the AJCC staging system.

All statistical analysis was performed using R (version 4.0.1; http://www.r-project.org/) software. The two-sided *p* < 0.05 was defined as statistically significant for tests in this study.

## Results

### Patient Characteristics

We extracted the data on 724 patients from the SEER database and randomly divided them into the development cohort and internal validation cohort. Another 70 patients were recruited from the West China Hospital of Sichuan University as the external validation cohort. The median follow-up of the development, the internal validation, and the external validation cohort are 67, 66, and 66 months, respectively. The characteristics are presented in **Table 1**.

**Table 1:** Characteristics of patients.

Variables	Development (*n* = 581)	Internal validation (*n* = 143)	External validation (*n* = 70)	*p*-value
**Age (%)**				
≤60	318 (54.7)	69 (48.3)	45 (64.3)	0.084
>60	263 (45.3)	74 (51.7)	25 (35.7)	
**Marital status (%)**				
Married	341 (58.7)	84 (58.7)	65 (92.9)	<0.001
Other	240 (41.3)	59 (41.3)	5 (7.1)	
**Race (%)**				
Black	77 (13.3)	18 (12.6)	0 (0.0)	<0.001
Other	27 (4.6)	6 (4.2)	70 (100.0)	
White	477 (82.1)	119 (83.2)	0 (0.0)	
**Histology (%)**				
Other	31 (5.3)	5 (3.5)	5 (7.1)	0.495
SCC	550 (94.7)	138 (96.5)	65 (92.9)	
**Grade (%)**				
≤II	457 (78.7)	112 (78.3)	55 (78.6)	0.996
>II	124 (21.3)	31 (21.7)	15 (21.4)	
**Site of Primary (%)**				
Body of penis	35 (6.0)	12 (8.4)	9 (12.9)	0.013
Glans penis	215 (37.0)	57 (39.9)	36 (51.4)	
Overlapping lesion	31 (5.3)	7 (4.9)	4 (5.7)	
Penis, NOS	228 (39.2)	50 (35.0)	10 (14.3)	
Prepuce	72 (12.4)	17 (11.9)	11 (15.7)	
**Size (%)**				
≤3	347 (59.7)	82 (57.3)	40 (57.1)	0.515
>5	118 (20.3)	26 (18.2)	11 (15.7)	
3 < T ≤ 5	116 (20.0)	35 (24.5)	19 (27.1)	
**Stage (%)**				
I	205 (35.3)	55 (38.5)	7 (10.0)	<0.001
II	227 (39.1)	53 (37.1)	35 (50.0)	
III	70 (12.0)	15 (10.5)	23 (32.9)	
IV	79 (13.6)	20 (14.0)	5 (7.1)	
**T (%)**				
T1	277 (47.7)	72 (50.3)	19 (27.1)	<0.001
T2	161 (27.7)	37 (25.9)	36 (51.4)	
T3	129 (22.2)	27 (18.9)	11 (15.7)	
T4	14 (2.4)	7 (4.9)	4 (5.7)	
**N (%)**				
N0	442 (76.1)	113 (79.0)	43 (61.4)	0.014
N+	139 (23.9)	30 (21.0)	27 (38.6)	
**M (%)**				
M0	561 (96.6)	139 (97.2)	69 (98.6)	0.637
M1	20 (3.4)	4 (2.8)	1 (1.4)	
**Surgery of Primary (%)**				
No	15 (2.6)	1 (0.7)	0 (0.0)	0.162
Yes	566 (97.4)	142 (99.3)	70 (100.0)	
**LND (%)**				
No	412 (70.9)	102 (71.3)	45 (64.3)	0.5
Yes	169 (29.1)	41 (28.7)	25 (35.7)	
**Radiotherapy (%)**				
No/Unknown	524 (90.2)	130 (90.9)	64 (91.4)	0.924
Yes	57 (9.8)	13 (9.1)	6 (8.6)	
**Chemotherapy (%)**				
No/Unknown	491 (84.5)	129 (90.2)	60 (85.7)	0.219
Yes	90 (15.5)	14 (9.8)	10 (14.3)	

The characteristics of patients in the development and internal validation cohorts are similar. Nearly half of the patients from the SEER database are not married (41.3%), but there is only 7% in the external validation cohort. Most of the patients from the SEER database have diseases in stages I and II; patients with stages III and IV are both less than 15%. However, in the external cohort, 32.9% of the patients have a stage III disease, more than twice as much as patients in the development and internal validation cohorts. In these three cohorts, more than 90% of patients have squamous cell carcinoma (SCC) of the penis, and there are a few patients who have distant metastases. Most of the patients received primary lesion surgery, but fewer patients received radiotherapy or chemotherapy.

### Determining the Predictors

**Figure 2:**
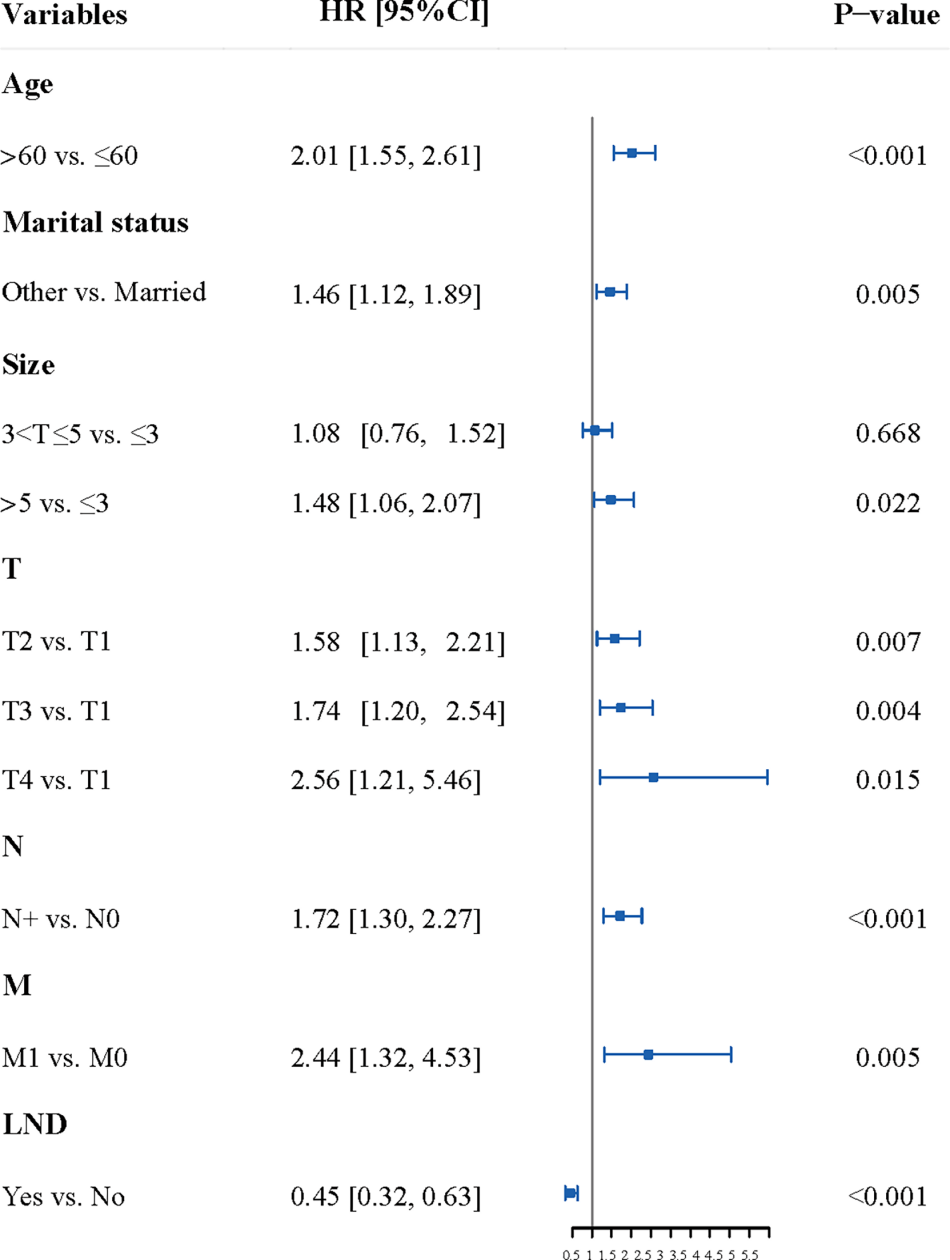
The Forest plot of multivariate analysis. The result of multivariate analysis showed that age, marital status, the size of the primary lesion, T, N, M, and the LND were the independent factors of the OS in patients with penile cancer. LND, the surgery of regional lymph node.

The results of multivariate analysis showed that age, marital status, the size of the primary lesion, T, N, M, and the LND were the independent factors of the OS in patients with penile cancer (**Table S1** and **Figure 2**).

### Developing and Validating the Nomogram

**Figure 3:**
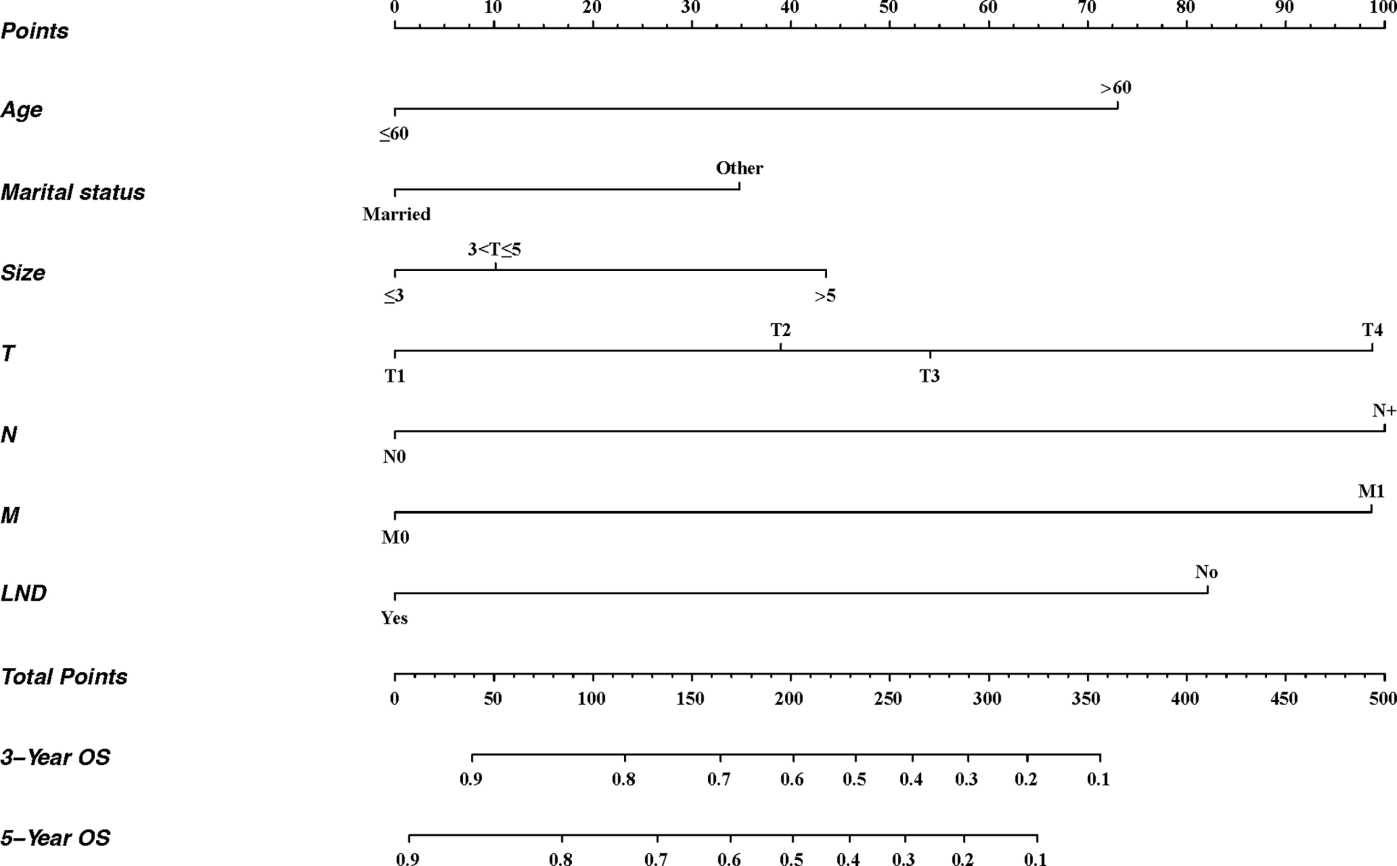
Nomogram for predicting 3- and 5-year OS of patients with penile cancer. For each patient, seven lines are drawn upward to determine the points received from the predictors in the nomogram. The sum of these points is located on the “Total Points” axis. Besides, two lines are drawn downward to determine the possibility of 3- and 5-year OS. LND, the surgery of regional lymph node.

Based on multivariate analysis, independent prognostic factors were included to establish a nomogram (**Figure 3**). There were seven lines drawn to determine the points from the predictors in the model, and the sum of these points could be located on the line of “Total Points”. Finally, the 3-year and 5-year overall survival rates could be calculated according to the “3-year OS” and “5-year OS” lines, respectively.

**Figure 4:**
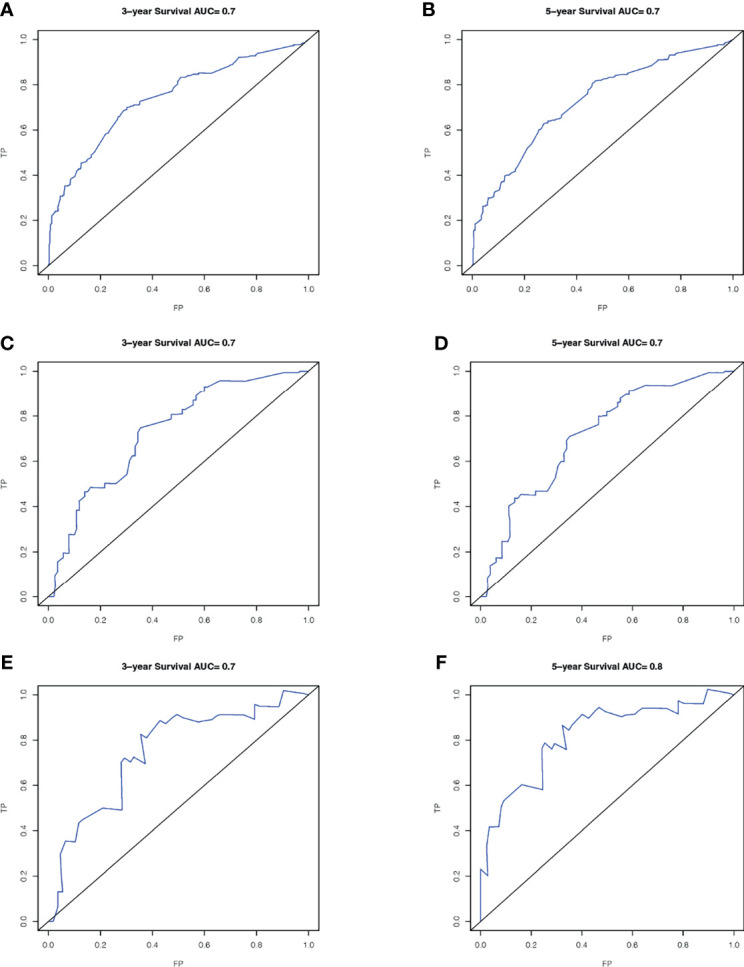
ROC curves of the nomogram in the prediction of prognosis. Three-year OS **(A)** and 5-year OS **(B)** in the development cohort; 3-year OS **(C)** and 5-year OS **(D)** in the internal validation cohort; 3-year OS **(E)** and 5-year OS **(F)** in the external validation cohort. ROC, receiver operating characteristic curve; AUC, areas under the ROC curve.

**Figure 5:**
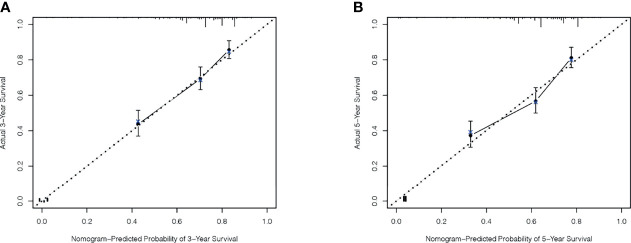
The calibration plots for predicting survival. Three-year OS **(A)** and 5-year OS **(B)** between the nomogram and the actual observation in the development cohort.

**Figure 6:**
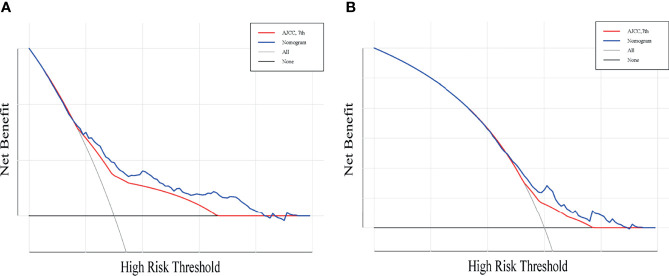
Decision curve analysis for the nomogram and AJCC stage in the prediction of prognosis of patients with penile cancer. The Decision curve analysis of the 3-year OS **(A)** and 5-year OS **(B)**. The *x*-axis shows the threshold probabilities, and the *y*-axis measures the net benefit calculated by adding the true positives and subtracting the false positives.

A total of 143 patients from the SEER database make up the internal validation cohort, and 70 patients from West China Hospital of Sichuan University constitute the external validation cohort to validate this predictive model. The 3- and 5-year AUCs were 0.7 and 0.7 in the development cohort (**Figures 4A, B**), 0.7 and 0.7 in the internal validation cohort (**Figures 4C, D**), and 0.7 and 0.8 in the external validation cohort (**Figures 4E, F**). The AUCs indicated good discrimination of this predictive model. The good agreement was confirmed by the calibration plots; the lines represented that the 3- and 5-year survival rates of the development (**Figures 5A, B**) were close to the ideal diagonal line. Besides, DCA plots showed that our predictive model had higher clinical effectiveness compared to the AJCC staging system (**Figure 6**).

**Figure 7:**
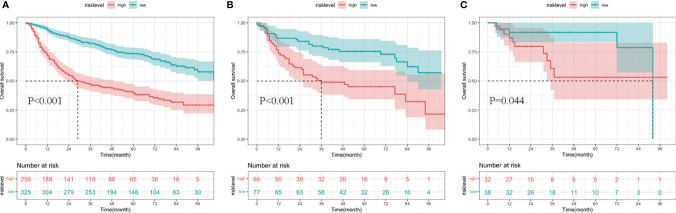
Kaplan–Meier curves of overall survival (OS) for patients in different risk levels. The survival of the low- and high-risk groups in the development cohort **(A)**, internal validation cohort **(B)**, and external validation cohort **(C)**.

Additionally, patients in the development and two validation cohorts were divided into two groups, respectively. The total points of patients were calculated; an optimal cutoff of the nomogram total score for survival was 172, with a sensitivity of 65.6% and a specificity of 68.8%. Patients whose scores were higher than 172 were classified into the high-risk group, and those whose risk scores were equal to or less than 172 were classified into the low-risk group. The survival analysis showed that compared to the low-risk group, the high-risk group had a significantly worse survival outcome in the training group (*p* < 0.001), internal validation group (*p* < 0.001), and external validation group (*p* = 0.044) (**Figure 7**).

## Discussion

Although the incidence of penile cancer is low, the advanced disease is associated with the considerable morbidity of patients. Small sample sizes and the lack of external validation limited the clinical use of existing models. Therefore, this study established and validated a novel prognostic model for clinical use.

In the current study, the results showed that marital status was the independent prognostic factor of penile cancer. The prognostic effect of marital status is largely ignored previously. The prognostic role of marriage was first exhibited by Mao et al. (23). In our study, we showed that marital status was an independent prognostic factor for penile cancer, and we included it as a vital predictor in the predictive model firstly. Besides the factors listed in Mao’s study, the possible reasons why marriage is associated with survival are as follows: (1) Married patients are more likely to be infected with HPV, which is mainly sexually transmitted (24). Although it increases the risk of penile cancer, positive HPV status may be correlated with a favorable prognosis (7). (2) Married patients are more likely to notice the penile lesion, so most of them may seek medical intervention at a relatively early stage of their illness. (3) Some researchers also mentioned that marriage positively correlated with a better outcome in other tumors that might be affected by psychological factors strongly (25, 26). This finding provides a theoretical basis for further research on the effect of marriage on the prognosis of penile cancer and indicates that more care should be provided for “other” patients.

Several models to predict survival in penile cancer have been established before. In 2006, two models were proposed by Kattan et al. to predict survival in penile cancer patients undergoing partial or total amputation (19). As the first study to develop the prognostic models for penile cancer, the sample size with 175 patients used to establish the models is considered insufficient. Besides, the extensibility of their study should be questioned as the 175 patients all came from Italy. Although the model presented relatively good discrimination, the above problems had limited the use of the model. In 2009, there is a model (27) based on the SEER stage and tumor grade in Zini’s study, but the SEER stage was verified to have yielded lower predictive accuracy compared with the AJCC stage and TNM stage in Thuret’s study (28). In 2015, Sun et al. developed and externally validated a predictive model for penile cancer (29). However, all the patients in the development and the external validation cohort of this study were treated in Europe, so the bias of model evaluation is inevitable because of the high homogeneity of the population. Besides, the vital prognostic role of therapy was ignored in Sun’s study. For patients who underwent regional lymph node dissection, a nomogram was proposed by Necchi et al. in 2019 (30). In Necchi’s study, the patients used to develop the model were from 7 centers in different regions, so it seems that Necchi’s model is more applicable for patients from different countries. However, similar to the studies of Kattan (19), Zini (27), Thuret (28), and Zheng (31), the applicability of models in different cohorts was not evaluated statistically by external validation. Thus, those studies should be interpreted with caution.

In this study, considering that OS is critical for patient counseling and decision-making, we developed a novel nomogram for penile cancer. Firstly, based on the large and comprehensive data in the SEER database, the number of patients in the development cohort used to develop the model was large, and this prognostic model relied on the combined effect of clinical, pathological, and therapeutic features that were easily available; thus, the simplicity and convenience of the predictive tool are the reason to adopt it in daily practice. Besides, the survival analysis of different risk levels presents a good risk stratification ability of this nomogram and is verified in the internal validation cohort from the SEER database and external validation from China. That is to say, this model is not only applicable to the American population, but also suitable for the Asian population, or at least the Chinese population.

This study also has limitations. Firstly, the sample size of the external validation cohort in this study is relatively small. Secondly, although SEER is an extremely valuable tool for clinical cancer research, the migration of patients in and out of SEER registry geographic catchment areas, the precision in registering the treatment modalities, and the unclear extension of lymphadenectomy in the SEER database may affect the results. In addition, some biological factors, such as PDL1 or HPV, are missing from the SEER database. However, this study is the first to use an Asian cohort to externally validate a model developed using a Western patient cohort. Of course, patients from outside Asia and the United States also need to be recruited in external validation cohorts to demonstrate better applicability of the model.

## Conclusion

This study develops a novel and clinically effective prognostic model for patients with penile cancer. The good performance of this model is determined by internal and external validation.

## AUTHOR’S NOTE

All statistical analyses were performed using R statistical software (version 4.0.1; http://www.r-project.org/). The code can be obtained by contacting the corresponding author, with reasonable requests.

## Data Availability Statement

The datasets presented in this article are not readily available because part of the data included in the study came from the single institution and was not publicly available. Requests to access the datasets should be directed to http://seer.cancer.gov/.

## Ethics Statement

This retrospective study complies with the ethical standards of the institution, and follows the principles of the Helsinki Declaration. Approval was granted by the Ethics Committee of West China Hospital of Sichuan University.

## Author Contributions

Conceptualization and Funding acquisition: XP. Methodology and Resources: XP, RL, and KC. Data curation: RL and KC. Software and Formal Analysis: RL, KC, and ZL. Supervision and Investigation: RL, ZW, and KC. Validation: RL, KC, and ZL. Visualization: RL, KC, and ZW. Writing—original draft and Writing—review and editing: RL and KC. Project administration: XP. All authors contributed to the article and approved the submitted version.

## Funding

The work was supported by the National Natural ScienceFoundation of China (82172842, 81803104, and 81672386), the Sichuan Province Science and Technology Support Program (2021YFSY008 and 2020YFS0276), the West China Nursing Discipline Development Special Fund Project (HXHL21008), the Technology Innovation Project of Chengdu Science and Technology Bureau (2019-YF05-00459-SN), the Postdoctoral Research and Development Fund and Translational Medicine Fund of West China Hospital (2020HXBH119 and CGZH19002), and the Sichuan Science and Technology Department Key Research and Development Project (22YFS0336).

## Conflict of Interest

The authors declare that the research was conducted in the absence of any commercial or financial relationships that could be construed as a potential conflict of interest.

## Publisher’s Note

All claims expressed in this article are solely those of the authors and do not necessarily represent those of their affiliated organizations, or those of the publisher, the editors and the reviewers. Any product that may be evaluated in this article, or claim that may be made by its manufacturer, is not guaranteed or endorsed by the publisher.

## Supplementary Material

Supplementary Table 1 Multivariate analysis to identify predictors of overall survival (OS). LND, the surgery of regional lymph node.

## Abbreviations

SEER, Surveillance, Epidemiology and End Results; LND, the surgery of regional lymph node; OS, overall survival; HPV, human papillomavirus; ILND, prophylactic inguinal lymphadenectomy; ICD-O-3, International Classification of Diseases for Oncology, Third Edition; AJCC, American Joint Committee on Cancer; ROC, receiver operating characteristic; AUC, the higher area under the ROC curve; DCA, decision curve analysis.
