# Supplementary material for: The Development and Validation of a Nomogram Incorporating Clinical, Pathological, and Therapeutic Features to Predict Overall Survival in Patients With Penile Cancer: A SEER-Based Study
Source: Front Oncol. 2022 Apr 5;12:840367. doi: 10.3389/fonc.2022.840367 (PMC9016192; doi:10.3389/fonc.2022.840367)
Supplement: Supplementary Table 1 — Multivariate analysis to identify predictors of overall survival (OS). LND, the surgery of regional lymph node. [file Table_1.docx]

Table S1 Multivariable Analyses to Identify Predictors.

|  | **HR[95%CI]** | **P-value** |
| --- | --- | --- |
| **Age** |  |  |
| ≤60 | reference |  |
| >60 | 2.01 [1.55, 2.61] | <0.001 |
| **Marital status** |  |  |
| Married | reference |  |
| Other | 1.46 [1.12, 1.89] | 0.005 |
| **Race** |  |  |
| Black | reference |  |
| Other | 1.10 [0.55, 2.19] | 0.797 |
| White | 1.01 [0.70, 1.45] | 0.966 |
| **Histology** |  |  |
| Other | reference |  |
| SCC | 1.31 [0.70, 2.44] | 0.398 |
| **Grade** |  |  |
| ≤II | reference |  |
| >II | 1.27 [0.93, 1.72] | 0.132 |
| **Primary** |  |  |
| Body of penis | reference |  |
| Glans penis | 1.02 [0.58, 1.82] | 0.938 |
| Overlapping lesion | 1.44 [0.69, 2.98] | 0.331 |
| Penis, NOS | 1.35 [0.77, 2.35] | 0.294 |
| Prepuce | 1.33 [0.68, 2.61] | 0.403 |
| **Size** |  |  |
| ≤3 | reference |  |
| 3<T≤5 | 1.08 [0.76, 1.52] | 0.668 |
| >5 | 1.48 [1.06, 2.07] | 0.022 |
| **T** |  |  |
| T1 | reference |  |
| T2 | 1.58 [1.13, 2.21] | 0.007 |
| T3 | 1.74 [1.20, 2.54] | 0.004 |
| T4 | 2.56 [1.21, 5.46] | 0.015 |
| **N** |  |  |
| N0 | reference |  |
| N+ | 1.72 [1.30, 2.27] | <0.001 |
| **M** |  |  |
| M0 | reference |  |
| M1 | 2.44 [1.32, 4.53] | 0.005 |
| **Surgery of Primary** |  |  |
| No | reference |  |
| Yes | 1.06 [0.48, 2.32] | 0.891 |
| **LND** |  |  |
| No | reference |  |
| Yes | 0.45 [0.32, 0.63] | <0.001 |
| **Radiotherapy** |  |  |
| No | reference |  |
| Yes | 1.19 [0.78, 1.82] | 0.426 |
| **Chemotherapy** |  |  |
| No | reference |  |
| Yes | 1.18 [0.79, 1.77] | 0.417 |
